# Construction of a prognostic model using lactylation-related genes for predicting the prognosis of glioma patients

**DOI:** 10.1097/MD.0000000000050273

**Published:** 2026-08-14

**Authors:** Jiajun Rao, Xiongwei Lu, Chenjun Luo, Zhao Zhang

**Affiliations:** a Department of Neurosurgery, People’s Hospital of Leshan, LeshanChina.

**Keywords:** Glioma, lactylation, predictive model, prognosis

## Abstract

Gliomas are the most lethal malignant tumors of the central nervous system, and their treatment continues to face serious challenges. Increasing evidence suggests that lactylation is strongly associated with tumorigenesis and progression. However, studies of lactylation in gliomas are rare. In this study, we screened for lactylation-related genes in glioblastoma affecting patient prognosis based on TCGA and GEO databases and constructed a prediction model for lactylation-related genes using various machine learning methods. In addition, the researchers have also performed tumor somatic mutation difference analysis, drug sensitivity analysis, and single-cell analysis. We developed a prognostic model for lactylation-related genes and validated its predictive power. Further analysis revealed differences in tumor somatic mutations between the high- and low-risk groups. We screened 50 drugs using drug sensitivity analysis, and at the single-cell level, we demonstrated the expression of characterized genes in glioblastoma. Our findings suggest that the lactylation-related gene prediction model can serve as a reliable tool for predicting the prognosis of patients with glioma.

## 1. Introduction

Glioblastoma, a primary malignant tumor of the intracranial region, is highly lethal and recurrent, with a 5-year survival rate of no more than 6% and a median survival of 14–16 months,^[1,2]^ even after aggressive treatment. Identifying effective molecular markers is crucial for glioblastoma treatment.

Lactate is considered both a waste product of cellular metabolism and, in recent years, researchers have revealed its multifaceted biological functions through a growing understanding of lactate. For example, lactate can serve as a source of energy that can be transported into cells, and it can also be involved in tumor angiogenesis, influencing the tumor microenvironment.^[3–6]^ Lactylation is a posttranslational modification related to lactate, which has been discovered in recent years. Lactylation occurs in cell membranes and organelles, participates in various cellular activities, and thus has biological functions. It has been found that lactylation can enhance macrophage polarization, promote immune escape of tumor cells, and promote tumor cell proliferation and metastasis.^[7–9]^ Targeting lactylation modification is a new and effective strategy for tumor therapy. There are a growing number of reports on the effects of lactylation on tumor progression. For example, in colorectal cancer, some studies found that GPR37 can promote liver metastasis by increasing the level of histone lactylation modification.^[10]^ Second, lactylation modifications can promote the expression of the autophagy enhancer protein RUBCNL, which in turn affects the resistance of colorectal cancer cells to bevacizumab.^[11]^ In bladder cancer, CircXRN2 inhibits histone lactylation-driven tumor progression through activation of the Hippo pathway.^[12]^ Wang et al found that BZW2 promotes the malignant progression of lung adenocarcinoma by promoting lactate production and lactylation of IDH3G.^[13]^

The role of lactylation modifications in glioblastoma is currently understudied. In this study, we collected previously published lactylation-related genes, compared multiple machine learning methods, and finally chose Random Forest to construct a prognostic model of glioblastoma with lactylation-related genes, which demonstrated a high predictive efficacy. We further conducted a tumor mutation load study and drug sensitivity analysis of the high-and low-lactylation groups. In addition, we centrally analyzed GSE84465 glioblastoma single-cell data. Notably, there have been no reports of lactylation-related genes constructing a prognostic model for glioblastoma, and our study fills this knowledge gap.

## 2. Materials and methods

### 2.1. Data collection

The UCSC Xena database (https://xena.ucsc.edu) yielded RNA sequencing and clinical information on 154 glioblastomas from The Cancer Genome Atlas (TCGA). A total of 1092 normal brain tissue samples were obtained from the Genotype-Tissue Expression (GTEx) project. RNA sequencing and sample information for 2 glioma datasets (GSE50161 and GSE43378) and 1 glioma single-cell dataset (GSE84465) were downloaded from GEO (https://www.ncbi.nlm.nih.gov/geo/). Information on age, IDH mutation status, and MGMT promoter status of glioblastoma patients in TCGA was obtained from Ceccarelli et al.^[14]^

### 2.2. Identification of lactylation-related glioblastoma-expressed differential genes

The R software “Limma” package was used to identify differentially expressed genes in 154 glioblastoma samples and 1092 normal brain samples from UCSC Xena. WGCNA was used to search for key genes of glioblastomas in the GSE50161 dataset to further explore the potential causative genes of glioblastomas. The lactylation gene set was obtained from Cheng et al,^[15]^ as previously published. Acquisition of 3 intersections defined as lactylation-related glioblastoma differential genes.

### 2.3. Screening of characterized genes for the construction of prognostic models

The lactylation-related glioblastoma differentially expressed genes were further screened using COX regression, LASSO, and random forest methods to obtain characteristic genes for constructing prognostic models.

### 2.4. Somatic mutations

Somatic mutation data for glioblastoma were downloaded from TCGA database, and waterfall plots were generated using the R package “Maftools.”

### 2.5. Drug sensitivity analysis

The “oncoPredict” R package was used to predict drug susceptibility in high and low-risk groups, with drug susceptibility files obtained from the Genomics of Drug Sensitivity in Cancer (GDSC) database (https://osf.io/c6tfx/).

### 2.6. Single-cell RNA analysis

The GSE84465 single-cell dataset comprised 4 human primary GBM samples. Analysis of the scRNA-seq data was conducted utilizing the “Seurat” package in R to eliminate low-quality cells based on mitochondrial gene expression levels. Data normalization was performed using the “NormalizeData” function, followed by filtering of the top 1500 highly variable genes using the “FindVariableFeatures” function. Principal component analysis (PCA) was subsequently applied to the 1500 selected genes using the “RunPCA” function. Cluster analysis was performed using the t-SNE method. Characterization scores were computed using the “AddModuleScore” function in the R package.

### 2.7. Statistical analysis

R software (version 4.4.0) was used for all statistical analyses. The Wilcoxon test was used to compare statistical differences between the 2 groups. *P*-value < 0.05 was considered statistically significant, and statistical significance is indicated by an asterisk (*). (* *P* < .05, ** *P* < .01, * *P* < .001, **** *P* < .0001).

## 3. Results

### 3.1. Identification of lactylation-related differential genes in glioblastoma

**Figure 1.**
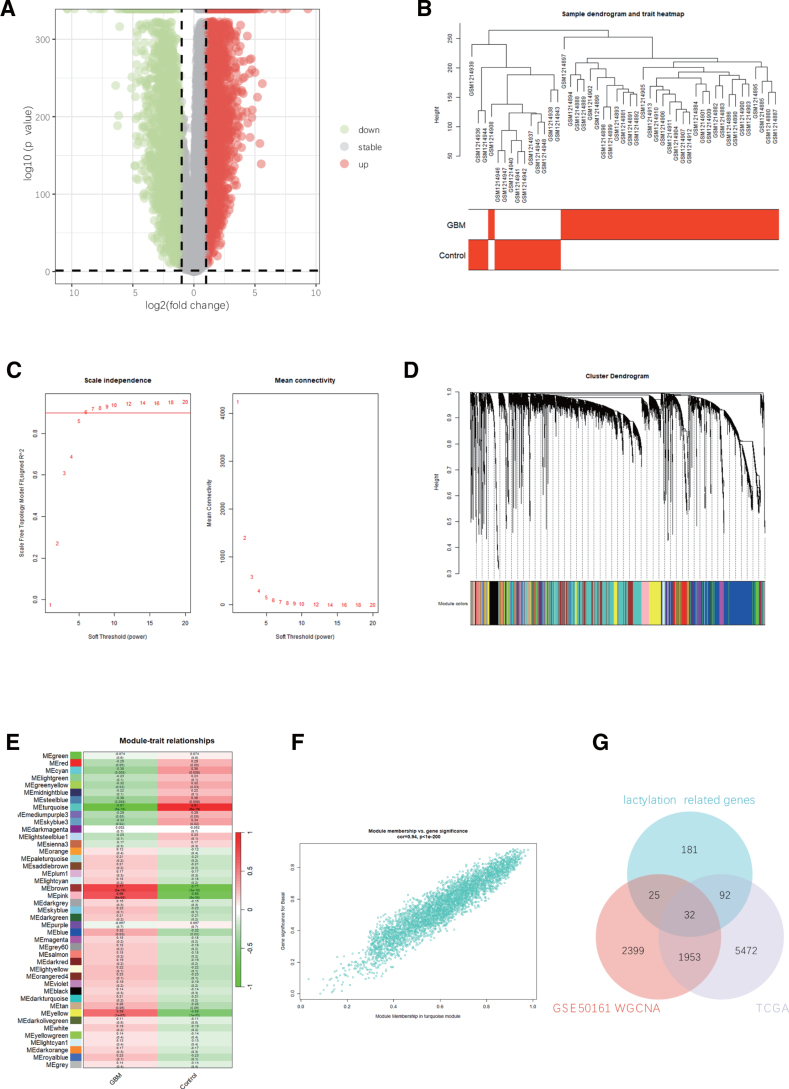
Identification of lactylation-related differential genes in glioblastoma: (A) Volcano plot showing differential expression up- or down-regulated in glioblastoma samples compared to normal brain tissue samples (green: down-regulated; red: up-regulated). (B) Cluster dendrogram of glioblastoma and normal control samples from WGCNA analysis. (C) Network Topology Analysis for Various Soft Thresholds. (D) Gene clustering based on differences in TOM, plotting clustering trees. (E) Heat map of modules concerning clinical shapes (F). The association between turquoise module membership and genetic importance is depicted in the scatter plot. (G) The intersection between 7549 differential genes, 4426 pivotal genes, and 332 lactylation-related genes.

We performed differential gene expression analysis on 154 glioblastoma samples from TCGA and 1092 normal brain tissue samples from GTEX. |logFC|>1 and adjusted *P* < .05 were considered significant for differential expression. We identified 7549 differentially expressed genes, and the results are shown in a volcano plot (Fig. 1A). WGCNA analysis was performed on glioblastoma patients and normal control individuals in the GSE50161 dataset to further identify the hub genes most associated with tumor traits. The results show that the network approached a scale-free distribution when β = 6. Subsequently, gene modules associated with clinical features were examined using a topological overlap matrix (TOM). Turquoise-colored modules were strongly associated with glioblastoma (Fig. 1B-F). Finally, 4426 genes were screened. Finally, we defined the intersection between 7549 differential genes, 4426 key genes, and 332 lactylation-related genes as lactylation-related differential genes (Fig. 1G).

### 3.2. Further screening of characterized genes using multiple machine learning

**Figure 2.**
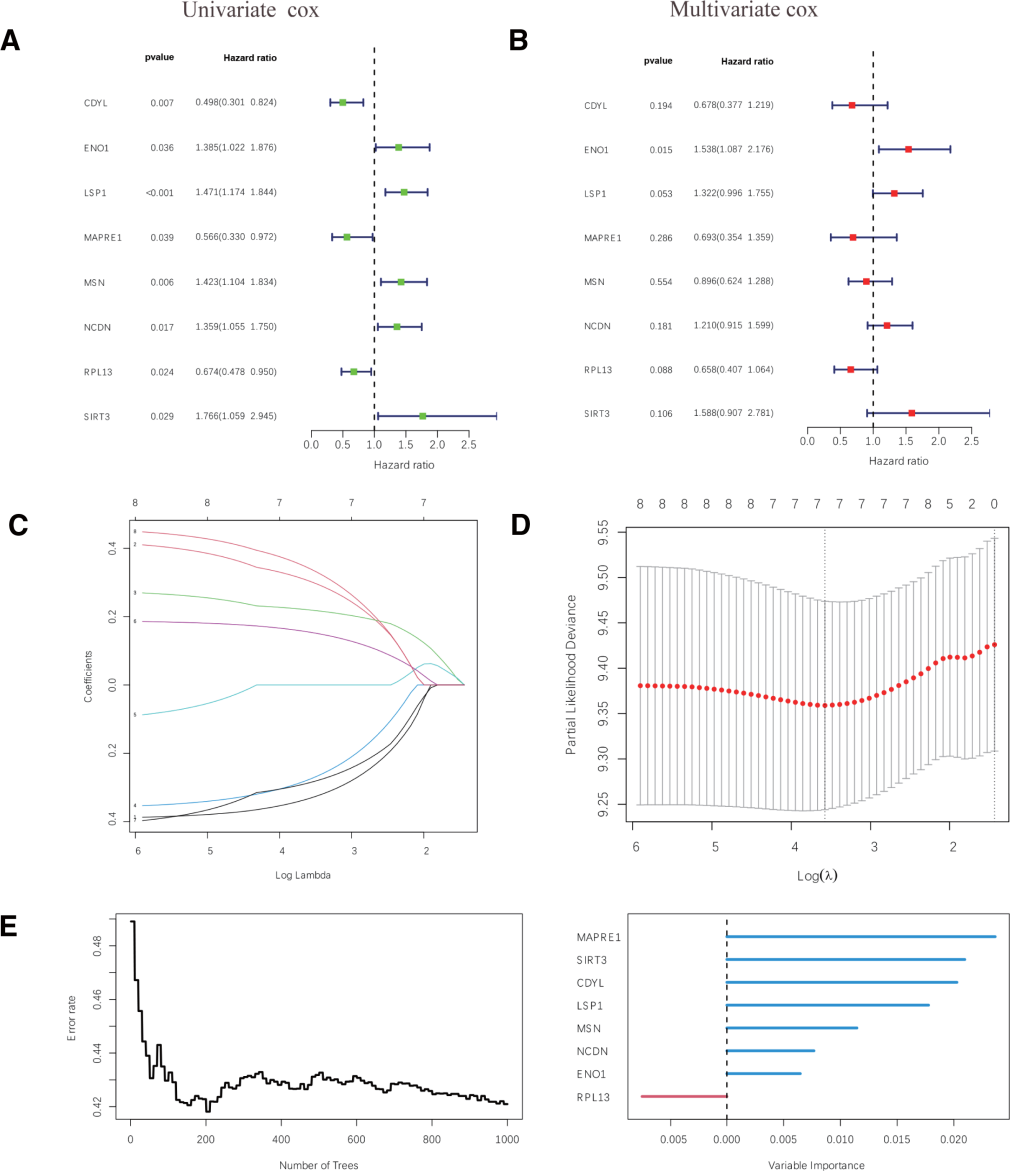
Screening of characterized genes using multiple machine learning methods:(A, B) Univariate and multivariate prognostic analyses. (C、D) lasso regression analysis. (E) Random forest analysis.

In the TCGA database, a total of 153 glioblastoma sample names were matched with their survival information. The TCGA cohort was used as the training set and GSE43378 as the validation set for subsequent studies. In previous work, we identified 32 differential genes related to lactylation. Through univariate Cox regression analysis, we further screened for genes associated with the prognosis of glioblastoma patients. Our analysis showed that 8 genes – CDYL, ENO1, LSP1, MAPRE1, MSN, NCDN, RPL13, and SIRT3 – are linked to glioblastoma prognosis (Fig. 2A). To obtain the optimal feature genes for constructing a prognostic model, we further analyzed these 8 genes using multivariate Cox regression analysis, LASSO analysis, and random forest analysis (Fig. 2B–E).

### 3.3. Comparison of the predictive efficacy of various machine learning constructed prognostic models

**Figure 3.**
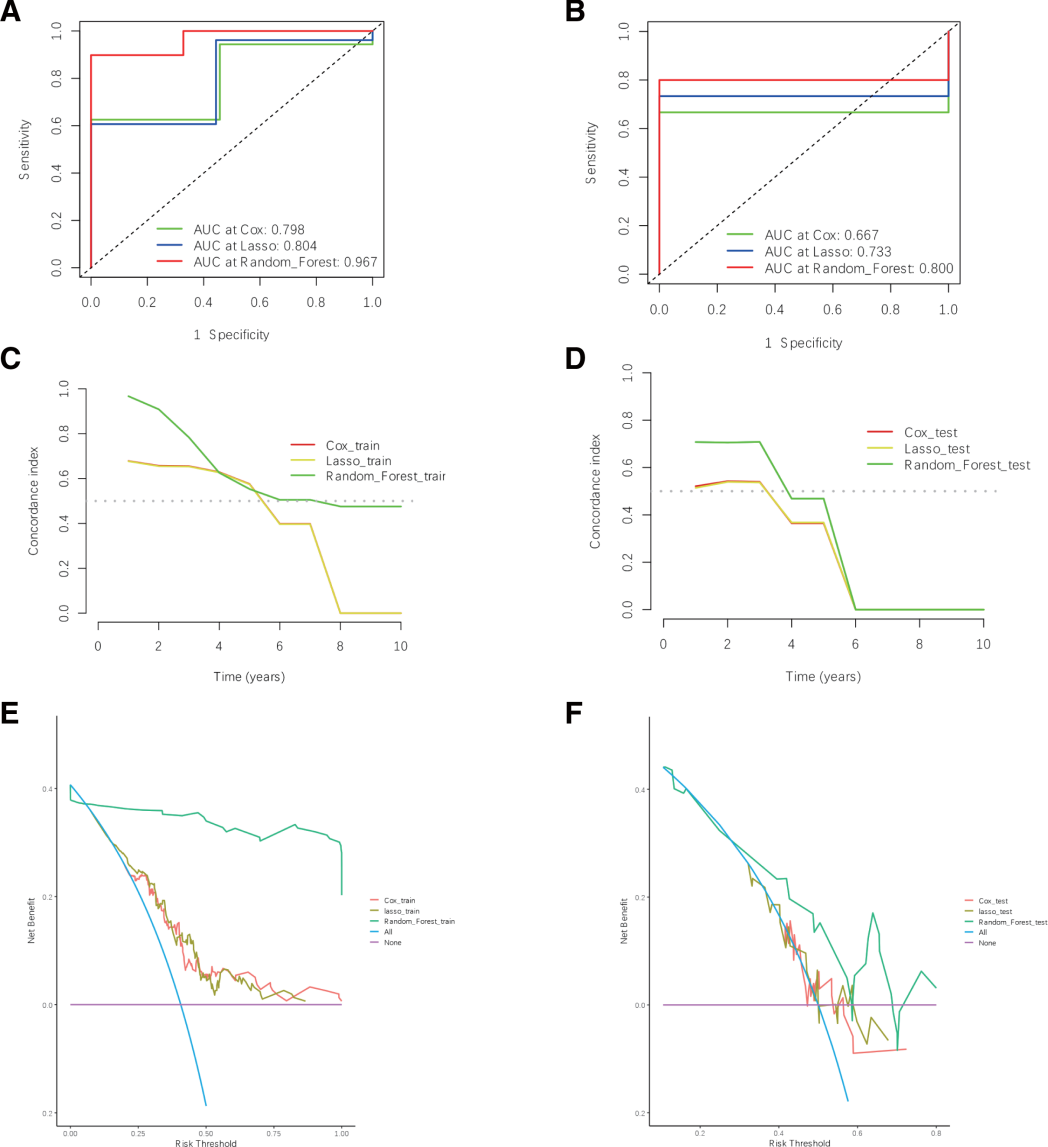
Comparison of various machine learning methods: (A) Comparison of 5-year time-dependent ROC curves for the training set; (B) Comparison of 5-year time-dependent ROC curves for the validation set;(C) Time C-index Comparison for the training set; (D) Time C-index Comparison for the validation set; (E) Comparison of decision curves for the training set (F) Comparison of decision curves for the validation set.

This study constructed a 5-year survival prognostic prediction model for glioblastoma based on Cox regression, LASSO, and random forest algorithms, and validated the model’s performance from multiple angles in the TCGA training set (Fig. 3A, 3C, 3E) and the GSE43378 validation set (Fig. 3B, 3D, 3F). ROC curve results showed that the AUC values for the Cox regression, LASSO analysis, and random forest models in the training set were 0.798, 0.804, and 0.967, respectively (Fig. 3A), while in the validation set, the AUC values were 0.667, 0.733, and 0.800, respectively (Fig. 3B). The random forest model outperformed both linear models in distinguishing outcomes across both datasets. Time-dependent C-index curves indicated that all 3 models could stably perform risk stratification over the 5-year follow-up period, with the random forest model maintaining the highest consistency index over the entire follow-up period (Figs. 3C, 3D). DCA decision curve analysis further confirmed that within a reasonable clinical risk threshold range, the random forest model offered a significantly higher net benefit than Cox regression or LASSO analysis (Fig. 3E, 3F). Taking into account its discriminative power, risk stratification stability, and clinical utility, the 5-year prognostic model for glioblastoma constructed using random forest exhibited the best overall predictive performance, followed by LASSO analysis, while traditional Cox regression showed relatively weaker predictive efficacy. The feature genes used to construct the model in the random forest algorithm were as follows: CDYL, ENO1, LSP1, MAPRE1, MSN, NCDN, RPL13, and SIRT3.

### 3.4. Prognostic differences between high and low risk groups and construction of a column chart using risk scores, clinical characteristics

**Figure 4.**
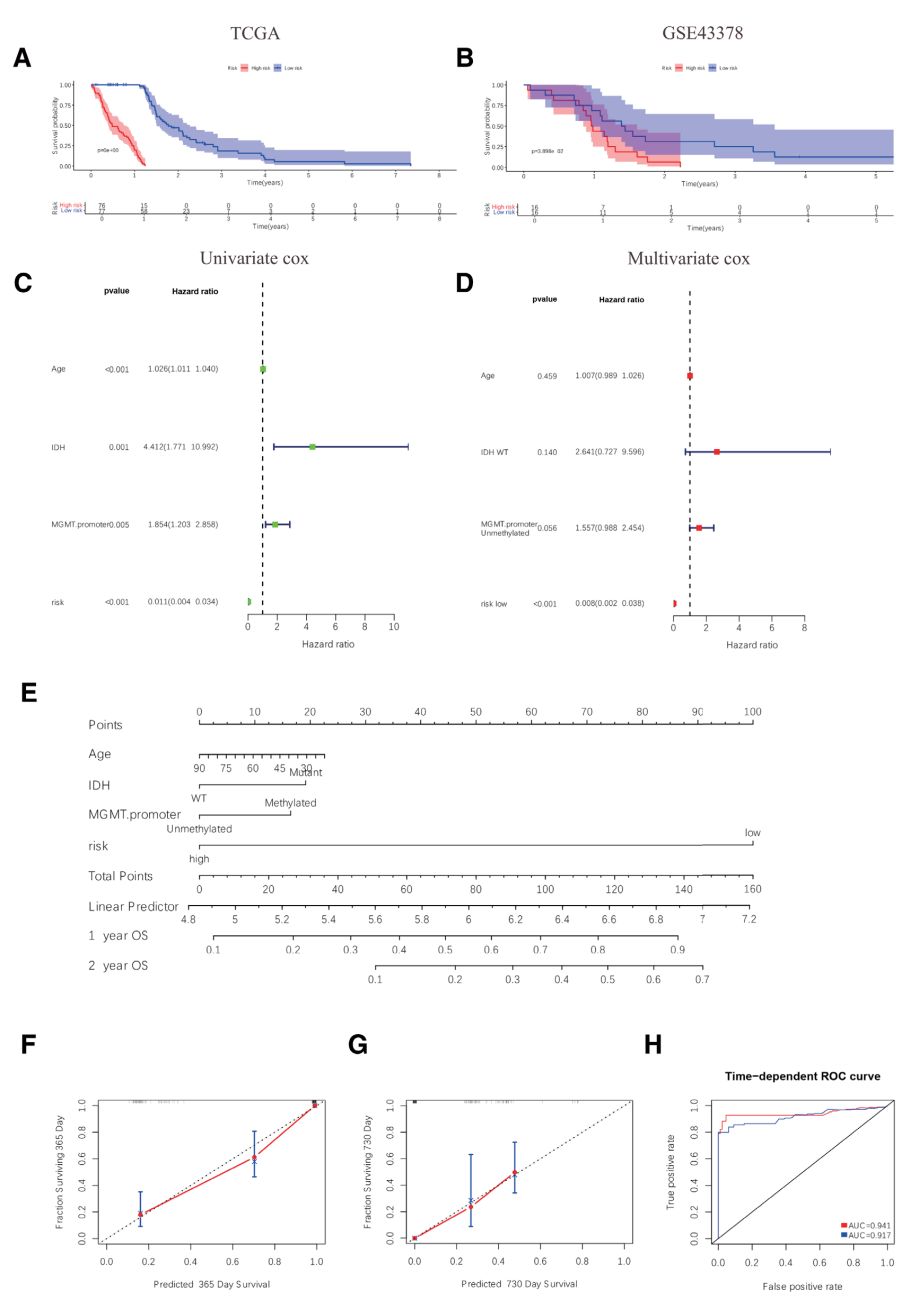
Prognostic value of risk scores combined with clinical characteristics for the overall survival in patients with glioblastoma: (A) Survival curves for high and low risk groups in TCGA; (B) Survival curves for high and low risk groups in GSE43378. (C, D) Univariate Cox regression and multivariate Cox regression were performed on 4 variables: risk score, age, IDH status, and MGMT promoter status. (E) Creation of a Nomogram to predict 1- and 2-year overall survival probabilities for glioblastoma patients in the TCGA cohort. (F, G) 1-year, 2-year calibration curves for line graphs. (H) ROC curves show the prognostic performance of the model.

The 153 glioblastoma patients in the training set were divided into a high-risk group (N = 76) and a low-risk group (N = 77) based on the median risk score value. Kaplan–Meier survival analysis showed that patients in the high-risk group had a poorer prognosis (Fig. 4A), a finding also confirmed in the validation set (Fig. 4B), indicating that the risk score effectively stratified patient survival. Univariate and multivariate Cox regression analyses confirmed that age, IDH mutation status, MGMT promoter methylation, and our risk score were all independent prognostic factors for overall survival in glioblastoma (Fig. 4C, 4D). A visual nomogram was constructed based on these independent predictors to estimate patients’ 1-year and 2-year overall survival probabilities (Fig. 4E). Calibration curves for the training set and external validation set indicated that the predicted probabilities from the nomogram were highly consistent with actual survival outcomes (Fig. 4F, 4G). Time-dependent ROC curves further verified that the model demonstrated stable and effective prognostic discrimination, with AUC values of 0.941 and 0.917 for 1-year and 2-year predictions, respectively (Fig. 4H). In summary, the nomogram model we built by combining multiple clinical features with the risk score showed excellent stratification performance, calibration, and discrimination, enabling accurate prediction of short-term survival outcomes in glioblastoma patients and offering high clinical value for prognostic assessment.

### 3.5. Somatic mutations and drug sensitivity analysis

**Figure 5.**
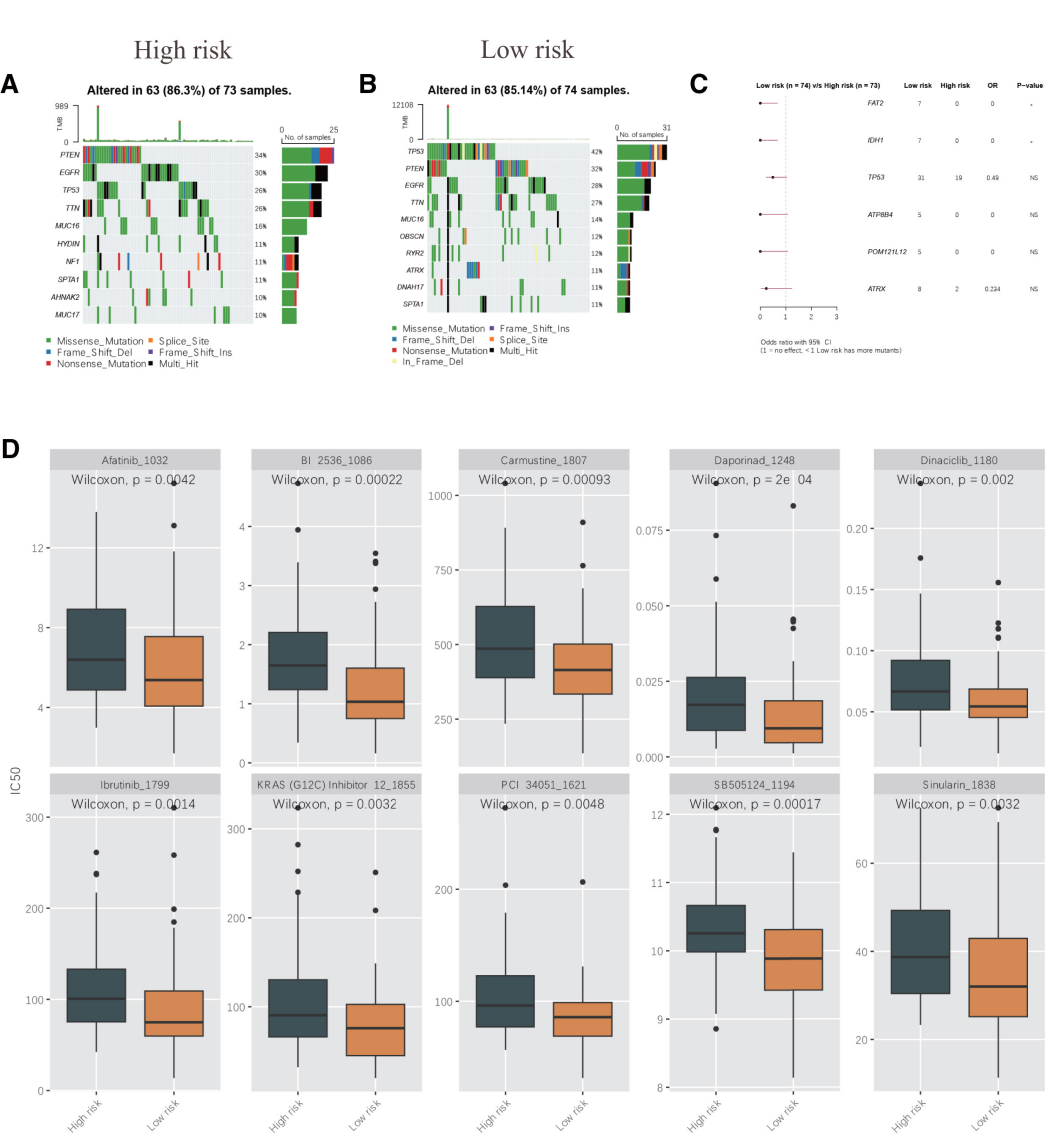
Analysis of somatic mutations and drug sensitivity in the high-risk and low-risk groups: The waterfall plots show the top 10 genes with the highest mutation frequencies in the high-risk (A) and low-risk (B) groups. (C) Mutationally differentiated genes in the high-risk and low-risk groups; (D) IC50 values of 10 drugs that differed between the high-risk and low-risk groups.

We examined tumor mutation profiles in subgroups of high-and low-risk groups for glioblastoma. Tumor mutations varied across the subgroups. The top 10 genes with the highest mutation frequencies in the high-risk group were PTEN, EGFR, TP53, TTN, MUC16, HYDIN, NF1, SPTA1, AHNAK2, and MUC17 (Fig. 5A), whereas the top 10 genes with the highest mutation frequencies in the low-risk group, in descending order, were TP53, PTEN, EGFR, TTN, MUC16, OBSCN, RYR2, ATRX, DNAH17, and SPTA1 (Fig. 5B). The mutated differential genes between the high- and low-risk groups were FAT2, IDH1(Fig. 5C). In addition, to further estimate the effect of the risk score on drug sensitivity, we compared the difference in the chemotherapy drug response (IC50) between the high- and low-risk groups (Fig. 5D). We screened 50 drugs that differed between the 2 groups, and the top 10 drugs with the smallest *p*-values were afatinib, BI 2536, carmustine, daporinad, dinaciclib, ibrutinib, KRAS (G12C) inhibitor 12, PCI 34051, SB505124, and sinularin. In the high-risk subgroup, the IC50 values for afatinib, BI 2536, carmustine, daporinad, dinaciclib, ibrutinib, KRAS (G12C) inhibitors 12, PCI 34051, SB505124, and sinularin were significantly higher than those in the low-risk group. The results suggest that the low-risk group responds better to this type of medication. In summary, the overall incidence of genomic mutations is similar between the high-risk and low-risk groups, but mutated genes and mutation patterns differ between the groups. Over ten antitumor drugs show significant differences in sensitivity between the 2 groups, and the 2 risk groups exhibit distinct response potentials to different drugs. This risk stratification strategy can be used to guide precision personalized medication in clinical practice.

### 3.6. Single-cell RNA analysis

**Figure 6.**
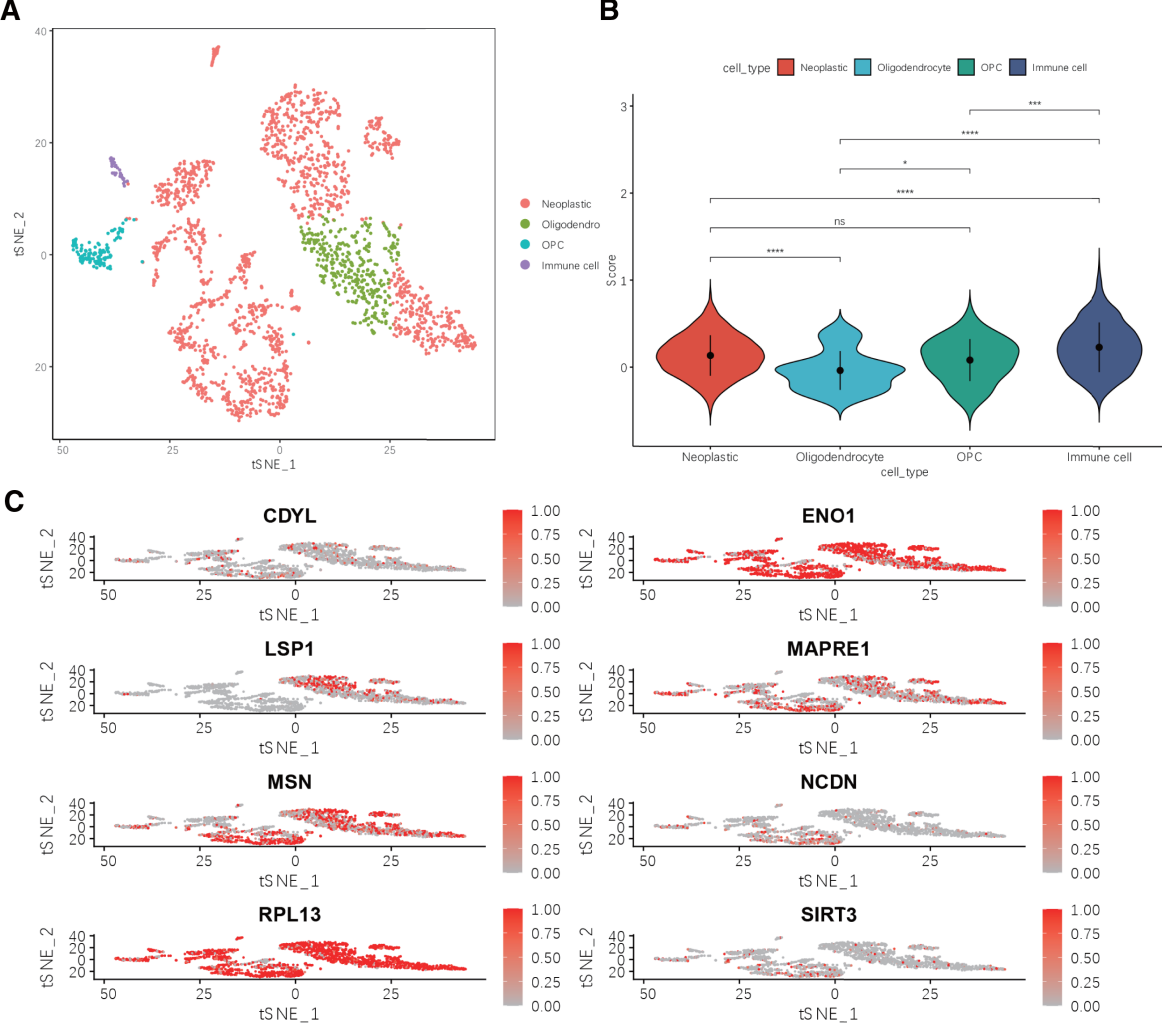
Single-cell RNA-seq analysis: (A) Cluster analysis of single-cell data using the t-SNE approach; (B) Expression of 8 lactylation-related signature genes in different cells of the tumor; (C)Scoring of lactation-associated genes in tumor tissues.

Glioblastoma single-cell data were reduced and clustered using Seurat. t-SNE reduced and clustered the tumor tissues into 13 clusters and annotated the cell types using the downloaded sample information (Fig. 6A). Expression distribution of 8 risk genes associated with emulsification in different cell types of glioblastomas (Fig. 6B). eno1, MAPRE1, and RPL13 were abundantly expressed in tumor cells, oligodendrocytes, OPC cells, and immune cells. LSP1 was mainly expressed in tumor cells and oligodendrocytes. AddModuleScore was applied to estimate the scores of 8 relevant risk genes in each cell type. The results showed that immune cells in tumor tissues scored the highest, followed by tumor cells and oligodendrocytes (Fig. 6C). This suggests our characterized genes may influence tumor progression by modulating the tumor microenvironment.

## 4. Discussion

Lactylation modification is a new posttranslational modification that has been extensively studied recently, and a large number of studies have demonstrated that lactylation modification is involved in the process of tumor development. It has been shown that the overall lactylation level of GSCs is significantly elevated compared to differentiated glioma cells and that lactylation contributes to the maintenance of glioma stem cells by PTBP1.^[16]^ Tumor resistance is an important factor for tumor progression and poor prognosis. Studies have shown that XRCC1 lactylation promotes ALDH1A3 overexpression and thus enhancing drug resistance in glioblastoma.^[17]^ In addition, Liu et al found that histone H3K9 lactylation can confer resistance to temozolomide in glioblastoma through retention of the MLH1 intron.^[18]^

However, to the best of our knowledge, prognostic models based on lactylation-related prognostic genes have not yet been reported for glioblastoma. In this study, we first screened lactylation-related prognostic genes and identified 8 featured genes: CDYL, ENO1, LSP1, MAPRE1, MSN, NCDN, RPL13, and SIRT3, using multiple machine learning methods. Recent studies have revealed that Chromodomain Y-like (CDYL) expression is upregulated in gliomas and that CDYL knockdown can inhibit glioma progression by affecting the polarization of CCL2-recruited monocytes/macrophages and M1-like TAMs in the tumor microenvironment.^[19]^ Enolase 1 (ENO1) functions as a glycolytic enzyme, fibrinogen receptor, and a DNA-binding protein. It is considered a potential target for treating various tumors.^[20–22]^ Zhou et al found that LSP1 regulated glioblastoma cell growth and migration by modulating the JAK2/STAT5 pathway.^[23]^ MAPRE1 can be used as a plasma biomarker for early colorectal cancer to complement current colorectal cancer screening methods.^[24]^ Moesin (MSN), a member of the ezrin-radixin-moesin family, accelerates colorectal cancer progression through the β-catenin-RUNX2 axis and has been identified as a potential therapeutic target for colorectal cancer.^[25]^ NCDN expression is upregulated in glioblastoma and is associated with patient Survival.^[26]^ RPL13, a 211 amino acid-long protein, is often thought to be associated with hypoplasia of the upper spinal epiphysis, leading to a short stature.^[27,28]^ SIRT3 plays an important role in various tumors.^[29,30]^ TCGA data were then used as the training set and GSE43378 as the validation set, which were categorized into high-risk and low-risk groups based on the median risk score. For the training set, the overall survival of glioblastoma patients in the low-risk group was significantly higher than that in the high-risk group. The results of the validation set are consistent with the training set. We performed univariate Cox analysis on the clinical characteristics of the risk score, age, IDH mutation status, and MGMT promoter methylation status. The results showed that the risk score, age, IDH mutation status, and MGMT promoter methylation status were all associated with the prognosis of glioblastoma patients. Multivariate COX regression analysis showed that the risk score was an independent indicator of the prognosis of patients with glioblastoma (*P* < .001). Subsequently, we constructed a nomogram combining the risk score and clinical features such as age, IDH mutation status, and MGMT promoter methylation status, which could reliably predict the prognosis of glioblastoma patients.

Tumor mutational burden (TMB) refers to the total number of somatic mutations occurring per megabase (Mb) in the tumor genome. By measuring the extent of genomic variation in tumor cells, this indirectly reflects the tumor cells’ ability to produce new antigens. Cytogenetics, somatic mutations, and copy number variations play a key role in disease progression. Innate drug resistance is generally considered to be caused by genetic mutations.^[31]^ For example, gene mutations in secondary and therapy-related acute myeloid leukemia not only affect sensitivity to chemotherapy drugs and drug resistance, and help predict patient prognosis, but can serve as effective therapeutic targets.^[32]^ We analyzed the differences in tumor somatic mutations between patients with glioblastoma in the high- and low-risk groups. The major mutational differences between the high- and low-risk groups were IDH1 and FAT2. IDH1 is an effective tumor prognostic marker for gliomas. It is an important guide for the grading of gliomas, and IDH1 mutations can inhibit the malignant phenotype of glioma cells and enhance drug sensitivity to temozolomide, radiotherapy.^[33–36]^ FAT2 encodes an atypical calreticulin involved in tissue remodeling and likely functions as a cell adhesion molecule to control cell proliferation.^[37]^ Rabé et al found that silencing of FAT2 affects glioblastoma cell survival and that FAT2 is associated with the acquisition of drug resistance in tumor cells.^[38]^ According to information in the TCGA database, alterations in FAT2 affect glioblastoma overall survival and disease-free/progression-free.^[39]^ Our study indicates that survival differences between the high- and low-risk groups may be linked to mutations in IDH1 and FAT2. In addition, drug sensitivity analyses showed that patients in different subgroups exhibited different responses to chemical and targeted drugs, with patients in the high-risk group showing a higher sensitivity to most drugs. These results suggest that risk scores can help in the search for effective therapeutic agents for glioblastoma.

The tumor microenvironment plays a big role in how tumors start and grow. For example, studies have reported elevated levels of pro-inflammatory cytokines in the blood of patients with pelvic inflammatory disease, which worsens the progression of prostate cancer and increases tumor aggressiveness.^[40]^ In this study, the cell types of tumor sample data in single-cell data were mainly annotated into 4 types: neoplastic cells, oligodendrocytes, OPC cells, and immune cells. The signature genes CDYL, ENO1, LSP1, MAPRE1, MSN, NCDN, RPL13, and SIRT3 were broadly expressed in the above- mentioned cell types, which were integrated into a gene set for further gene set scoring analysis, and showed that immune cells, neoplastic cells, oligodendrocytes, and OPC cells all scored higher, which reinforced the role of the signature genes.

## 5. Conclusions

In summary, our study constructed a prognostic model for glioblastoma using 8 lactylation-related genes, which demonstrated high accuracy in predicting the prognosis of glioblastoma patients. The risk score was closely associated with tumor mutational burden and drug sensitivity. The study further revealed the expression of these 8 lactylation genes across different cell types in a single-cell dataset of glioblastoma, along with the enrichment scores of these gene sets. This indicates that the characteristic genes may play important roles through the tumor microenvironment. Our research provides new insights into the diagnosis and treatment of glioblastoma. Lactylation-related genes could become new prognostic markers for glioblastoma. This study has some limitations. Our findings lack further experimental validation, which will be addressed in the next step.

## Author contributions

**Data curation:** Jiajun Rao, Chenjun Luo.

**Writing – original draft:** Jiajun Rao.

**Visualization:** Xiongwei Lu.

**Writing – review & editing:** Zhao Zhang.

## References

[1] Omuro A DeAngelis LM. Glioblastoma and other malignant gliomas: a clinical review. JAMA. 2013;310:1842–50. 24193082 10.1001/jama.2013.280319

[2] Ohgaki H Kleihues P. Population-based studies on incidence, survival rates, and genetic alterations in astrocytic and oligodendroglial gliomas. J Neuropathol Exp Neurol. 2005;64:479–89. 15977639 10.1093/jnen/64.6.479

[3] Sonveaux P Végran F Schroeder T. Targeting lactate-fueled respiration selectively kills hypoxic tumor cells in mice. J Clin Invest. 2008;118:3930–42. 19033663 10.1172/JCI36843 PMC2582933

[4] Rabinowitz JD Enerbäck S. Lactate: the ugly duckling of energy metabolism. Nat Metab. 2020;2:566–71. 32694798 10.1038/s42255-020-0243-4 PMC7983055

[5] Végran F Boidot R Michiels C Sonveaux P Feron O. Lactate influx through the endothelial cell monocarboxylate transporter MCT1 supports an NF-κB/IL-8 pathway that drives tumor angiogenesis. Cancer Res. 2011;71:2550–60. 21300765 10.1158/0008-5472.CAN-10-2828

[6] Certo M Tsai C-H Pucino V Ho P-C Mauro C. Lactate modulation of immune responses in inflammatory versus tumour microenvironments. Nat Rev Immunol. 2021;21:151–61. 32839570 10.1038/s41577-020-0406-2

[7] Hagihara H Shoji H Otabi H. Protein lactylation induced by neural excitation. Cell Rep. 2021;37:109820. 34644564 10.1016/j.celrep.2021.109820

[8] Gu J Zhou J Chen Q. Tumor metabolite lactate promotes tumorigenesis by modulating MOESIN lactylation and enhancing TGF-β signaling in regulatory T cells. Cell Rep. 2022;39:110986. 35732125 10.1016/j.celrep.2022.110986

[9] Zhang Q Cao L Xu K. Role and mechanism of lactylation in cancer. Zhongguo Fei Ai Za Zhi. 2024;27:471–9. 39026499 10.3779/j.issn.1009-3419.2024.102.20 PMC11258650

[10] Zhou J Xu W Wu Y. GPR37 promotes colorectal cancer liver metastases by enhancing the glycolysis and histone lactylation via Hippo pathway. Oncogene. 2023;42:3319–30. 37749229 10.1038/s41388-023-02841-0

[11] Li W Zhou C Yu L. Tumor-derived lactate promotes resistance to bevacizumab treatment by facilitating autophagy enhancer protein RUBCNL expression through histone H3 lysine 18 lactylation (H3K18la) in colorectal cancer. Autophagy. 2024;20:114–30. 37615625 10.1080/15548627.2023.2249762 PMC10761097

[12] Xie B Lin J Chen X. CircXRN2 suppresses tumor progression driven by histone lactylation through activating the Hippo pathway in human bladder cancer. Mol Cancer. 2023;22:151. 37684641 10.1186/s12943-023-01856-1 PMC10486081

[13] Wang M He T Meng D. BZW2 modulates lung adenocarcinoma progression through glycolysis-mediated IDH3G lactylation modification. J Proteome Res. 2023;22:3854–65. 37955350 10.1021/acs.jproteome.3c00518

[14] Ceccarelli M Barthel FP Malta TM.; TCGA Research Network. Molecular profiling reveals biologically discrete subsets and pathways of progression in diffuse glioma. Cell. 2016;164:550–63. 26824661 10.1016/j.cell.2015.12.028 PMC4754110

[15] Cheng Z Huang H Li M Liang X Tan Y Chen Y. Lactylation-related gene signature effectively predicts prognosis and treatment responsiveness in hepatocellular carcinoma. Pharmaceuticals (Basel). 2023;16:644. 37242427 10.3390/ph16050644 PMC10221268

[16] Zhou Z Yin X Sun H. PTBP1 lactylation promotes glioma stem cell maintenance through PFKFB4-driven glycolysis. Cancer Res. 2025;85:739–57. 39570804 10.1158/0008-5472.CAN-24-1412

[17] Li G Wang D Zhai Y. Glycometabolic reprogramming-induced XRCC1 lactylation confers therapeutic resistance in ALDH1A3-overexpressing glioblastoma. Cell Metab. 2024;36:1696–710.e10. 39111285 10.1016/j.cmet.2024.07.011

[18] Yue Q Wang Z Shen Y. Histone H3K9 lactylation confers temozolomide resistance in glioblastoma via LUC7L2-mediated MLH1 intron retention. Adv Sci (Weinh). 2024;11:e2309290. 38477507 10.1002/advs.202309290 PMC11109612

[19] Kong JG Mei Z Zhang Y Xu LZ Zhang J Wang Y. CDYL knockdown reduces glioma development through an antitumor immune response in the tumor microenvironment. Cancer Lett. 2023;567:216265. 37302564 10.1016/j.canlet.2023.216265

[20] Qiao G Wu A Chen X Tian Y Lin X. Enolase 1, a moonlighting protein, as a potential target for cancer treatment. Int J Biol Sci. 2021;17:3981–92. 34671213 10.7150/ijbs.63556 PMC8495383

[21] Ma Q Jiang H Ma L. The moonlighting function of glycolytic enzyme enolase-1 promotes choline phospholipid metabolism and tumor cell proliferation. Proc Natl Acad Sci U S A. 2023;120:e2209435120. 37011206 10.1073/pnas.2209435120 PMC10104498

[22] Fu QF Liu Y Fan Y. Alpha-enolase promotes cell glycolysis, growth, migration, and invasion in non-small cell lung cancer through FAK-mediated PI3K/AKT pathway. J Hematol Oncol. 2015;8:22. 25887760 10.1186/s13045-015-0117-5 PMC4359783

[23] Cong P Hou HY Wei W Zhou Y Yu XM. MiR-920 and LSP1 co-regulate the growth and migration of glioblastoma cells by modulation of JAK2/STAT5 pathway. J Bioenerg Biomembr. 2020;52:311–20. 32770294 10.1007/s10863-020-09848-2

[24] Taguchi A Rho JH Yan Q. MAPRE1 as a plasma biomarker for early-stage colorectal cancer and adenomas. Cancer Prev Res (Phila). 2015;8:1112–9. 26342024 10.1158/1940-6207.CAPR-15-0077 PMC4633385

[25] Huang CY Wei PL Batzorig U Makondi PT Lee CC Chang YJ. Identification of Moesin (MSN) as a potential therapeutic target for colorectal cancer via the β-Catenin-RUNX2 Axis. Int J Mol Sci . 2023;24:10951–10951. 37446127 10.3390/ijms241310951 PMC10341927

[26] Huang X Xu C Dai H. NCDN is a potential biomarker and therapeutic target for glioblastoma. J Cancer. 2024;15:1067–76. 38230206 10.7150/jca.90535 PMC10788732

[27] Le Caignec C Ory B Lamoureux F. RPL13 variants cause spondyloepimetaphyseal dysplasia with severe short stature. Am J Hum Genet. 2019;105:1040–7. 31630789 10.1016/j.ajhg.2019.09.024 PMC6849359

[28] Jacob P Lindelöf H Rustad CF. Clinical, genetic and structural delineation of RPL13-related spondyloepimetaphyseal dysplasia suggest extra-ribosomal functions of eL13. NPJ Genom Med. 2023;8:39. 37993442 10.1038/s41525-023-00380-x PMC10665555

[29] Ouyang S Zhang Q Lou L. The double-edged sword of SIRT3 in cancer and its therapeutic applications. Front Pharmacol. 2022;13:871560. 35571098 10.3389/fphar.2022.871560 PMC9092499

[30] Torrens-Mas M Oliver J Roca P Sastre-Serra J. SIRT3: oncogene and tumor suppressor in cancer. Cancers (Basel). 2017;9:90. 28704962 10.3390/cancers9070090 PMC5532626

[31] Ramisetty S Kulkarni P Bhattacharya S. A Systems biology approach for addressing cisplatin resistance in non-small cell lung cancer. J Clin Med. 2023;12:599. 36675528 10.3390/jcm12020599 PMC9861808

[32] Goel H Rahul E Gupta I. Molecular and genomic landscapes in secondary & therapy related acute myeloid leukemia. Am J Blood Res. 2021;11:472–97. 34824881 PMC8610791

[33] Lin L Cai J Tan Z. Mutant IDH1 Enhances Temozolomide Sensitivity via Regulation of the ATM/CHK2 Pathway in Glioma. Cancer Res Treat. 2021;53:367–77. 33070553 10.4143/crt.2020.506 PMC8053882

[34] Han X Zhou H Sun W. IDH1(R132H) mutation increases radiotherapy efficacy and a 4-gene radiotherapy-related signature of WHO grade 4 gliomas. Sci Rep. 2023;13:19659. 37952042 10.1038/s41598-023-46335-1 PMC10640646

[35] Cui D Ren J Shi J. R132H mutation in IDH1 gene reduces proliferation, cell survival and invasion of human glioma by downregulating Wnt/β-catenin signaling. Int J Biochem Cell Biol. 2016;73:72–81. 26860959 10.1016/j.biocel.2016.02.007

[36] Juratli TA Cahill DP McCutcheon IE. Determining optimal treatment strategy for diffuse glioma: the emerging role of IDH mutations. Expert Rev Anticancer Ther. 2015;15:603–6. 25980633 10.1586/14737140.2015.1047351 PMC5089964

[37] Fulford AD McNeill H. Fat/Dachsous family cadherins in cell and tissue organisation. Curr Opin Cell Biol. 2020;62:96–103. 31739265 10.1016/j.ceb.2019.10.006

[38] Rabé M Fonteneau L Oliver L. Cellular heterogeneity and cooperativity in glioma persister cells under temozolomide treatment. Front Cell Dev Biol. 2022;10:835273. 35693929 10.3389/fcell.2022.835273 PMC9174429

[39] Rabé M Dumont S Álvarez-Arenas A. Identification of a transient state during the acquisition of temozolomide resistance in glioblastoma. Cell Death Dis. 2020;11:19. 31907355 10.1038/s41419-019-2200-2 PMC6944699

[40] Chakravarty D Ratnani P Huang L. Association between incidental pelvic inflammation and aggressive prostate cancer. Cancers (Basel). 2022;14:2734. 35681714 10.3390/cancers14112734 PMC9179284

